# Role of pannexin-1 in the cellular uptake, release and hydrolysis of anandamide by T84 colon cancer cells

**DOI:** 10.1038/s41598-019-44057-x

**Published:** 2019-05-20

**Authors:** Mireille Alhouayek, René Sorti, Jonathan D. Gilthorpe, Christopher J. Fowler

**Affiliations:** 1 Department of Pharmacology and Clinical Neuroscience, Umeå University, SE-901 87 Umeå, Sweden; 2 Present Address: Bioanalysis and Pharmacology of Bioactive Lipids Research Group, Louvain Drug Research Institute, Université catholique de Louvain, B1.72.01-1200 Bruxelles, Belgium

**Keywords:** Lipids, Preclinical research

## Abstract

The large pore ion channel pannexin-1 (Panx1) has been reported to play a role in the cellular uptake and release of anandamide (AEA) in the hippocampus. It is not known whether this is a general mechanism or limited to the hippocampus. We have investigated this pharmacologically using T84 colon cancer cells. The cells expressed Panx1 at the mRNA level, and released ATP in a manner that could be reduced by treatment with the Panx1 inhibitors carbenoxolone and mefloquine and the Panx1 substrate SR101. However, no significant effects of these compounds upon the uptake or hydrolysis of exogenously applied AEA was seen. Uptake by T84 cells of the other main endocannabinoid 2-arachidonoylglycerol and the AEA homologue palmitoylethanolamide was similarly not affected by carbenoxolone or mefloquine. Total release of tritium from [^3^H]AEA-prelabelled T84 cells over 10 min was increased, rather than inhibited by carbenoxolone and mefloquine. Finally, AEA uptake by PC3 prostate cancer and SH-SY5Y neuroblastoma cells, which express functional Panx1 channels, was not inhibited by carbenoxolone. Thus, in contrast to the hippocampus, Panx1 does not appear to play a role in AEA uptake and release from the cells studied under the conditions used.

## Introduction

The endocannabinoid system comprises the G-protein coupled CB_1_ and CB_2_ receptors, the endogenous ligands anandamide (AEA) and 2-arachidonoylglycerol (2-AG) and their synthetic and metabolic enzymes. It acts as an “on demand” regulatory system in the brain as well as in the periphery^1^. One important example is in the gut. In experimental colitis, activation of CB receptors, either directly with agonists, or indirectly by preventing the hydrolysis of the endocannabinoids, has beneficial effects^2–6^. Conversely, mice lacking CB receptors show increased susceptibility to experimental colitis^7^. AEA is released together with other *N*-acylethanolamines, such as the anti-inflammatory compound palmitoylethanolamide (PEA), and PEA can also influence gut function^8–10^. In humans, there is reduced expression of *N*-acylphosphatidylethanolamine-phospholipase D, a key enzyme in the synthesis of AEA and PEA in the colonic epithelium at the onset of ulcerative colitis^11,12^. Together, these data point to a protective role of the endocannabinoid system and of PEA in the gut and the potential of blockade of the metabolism of 2-AG, AEA and related *N*-acylethanolamines for the treatment of ulcerative colitis.

The large pore ion channel pannexin 1 (Panx1) has also been implicated in gut dysfunction. These channels, which are ubiquitous in mammalian tissues, are non-selective and mediate the release of small molecules including ATP^13^ and epoxyeicosatrienoic acids^14^ (review, see^15^). Using immunolabelling, relative expression of both cell-surface and intracellular Panx1 have been reported to vary in individual cells in culture, presumably reflecting both the life cycle of this protein and its potential different cellular roles^16^. Panx1 is involved in inflammation-induced enteric neuron death^17^, and in a variety of colon cancer cell lines, ligands for liver X receptors produced cell death by a Panx1-dependent mechanism^18^. In humans, Panx1 immunoreactivity is found in the enteric ganglia, epithelial and goblet cells, blood vessel endothelium and in erythrocytes^19^. Expression of Panx1 protein is reduced in the colonic muscularis mucosa and in the enteric neurons of patients with Crohn’s disease, but not in ulcerative colitis^17,18^.

AEA, PEA and 2-AG are cleared from the extracellular space by cellular uptake followed by intracellular catabolism^20^. The enzymes involved in the catabolism of these lipids have been well characterised. Thus, AEA is hydrolysed to arachidonic acid primarily by fatty acid amide hydrolase (FAAH) but is also a substrate for *N*-acylethanolamine acid amidase (NAAA) and can also be oxidatively metabolised by cyclooxygenase-2 and members of the P450 enzyme family. PEA is metabolised by both NAAA and FAAH, whilst 2-AG can be metabolised both by monoacylglycerol lipase and by other hydrolytic and oxidative enzymes^21^.

In contrast to the well-characterised enzymatic pathways for endocannabinoid and PEA catabolism, the mechanism(s) by which these lipids are accumulated by cells has been a matter of controversy. In many cells AEA uptake is regulated by FAAH activity (by regulating the intra-: extracellular gradient of this ligand)^22^. Further, proteins such as fatty acid binding proteins, heat shock proteins, albumin and possibly sterol carrier protein-2 can act as intracellular carriers for AEA^23–25^, and in endothelial cells, the uptake of the fluorescent AEA analogue SKM 4-45-1 is dependent upon the expression level of TRPV1 ion channels^26^. However, the existence (or lack thereof) of a cell surface endocannabinoid transporter protein has been a bone of contention (reviews see^27,28^).

Over the last few years Drs. Matt Hill, Roger Thompson and colleagues have presented data suggesting that in hippocampal pyramidal neurons, AEA can be transported by Panx1^29,30^. These authors took advantage of the fact that AEA, in addition to its effects upon CB_1_ receptors, can activate TRPV1 ion channels by binding to an intracellular location on this protein. They showed that a downstream effect of TRPV1 (glutamate release) was induced by administration of extracellular AEA in a manner blocked by administration of an anti-Panx1 antibody. Further, AEA levels in hippocampal slices were increased by blocking Panx1 channels with ^10^panx, a peptide blocker of Panx1. The authors also showed that flux of the Panx1 permeable dye sulforhodamine 101 (SR101) was reduced by 50 µM AEA, i.e. that AEA competed with the dye, and that transfection of HEK293T cells with Panx1 increased their level of uptake of SKM 4-45-1. Finally, they also showed that the transport through Panx1 channels was bidirectional, since postsynaptic loading with AEA increased TRPV1 signalling in a manner blocked with αPanx1^30^. These data are elegant and raise the question as to whether it is a general phenomenon in all cells naturally expressing Panx1 channels or whether it is restricted to pyramidal neurones alone; additionally as to whether Panx1 can act as an intracellular carrier for AEA akin to that seen for fatty acid binding proteins^23^. These questions are not facile: if Panx1 gates the uptake of AEA into gut epithelial cells and enteric neurons, then a Panx1 inhibitor could have duplicate beneficial effects in both potentiating extracellular AEA levels and preventing Panx1-mediated deleterious effects.

To our knowledge, the only available data on the relationship between Panx1 and AEA other than the study of^30^ has been in mouse taste buds, where taste stimuli-mediated cell-to-cell communication is mediated by Panx1 channels. However, this communication is not prevented at all by 50 µM AEA^31^, in contrast to the situation in the hippocampus^30^. Nothing is known about the role of naturally-occurring Panx1 channels in mediating cellular AEA accumulation in peripheral cells. Given the importance of AEA, PEA, 2-AG and Panx1 in the gut, we have investigated the effect of pharmacological inhibition of Panx1 upon the uptake, release and hydrolysis of AEA, and upon the uptake of PEA and 2-AG into human T84 colon cancer cells. In addition, we have investigated whether or not Panx1 inhibition affects the uptake of AEA into human PC3 prostate cancer cells and into both undifferentiated and differentiated SH-SY5Y neuroblastoma cells, since these are known to express functionally active Panx1 channels^32,33^.

## Results

### Expression of panx1 in T84 cells

**Figure 1:**
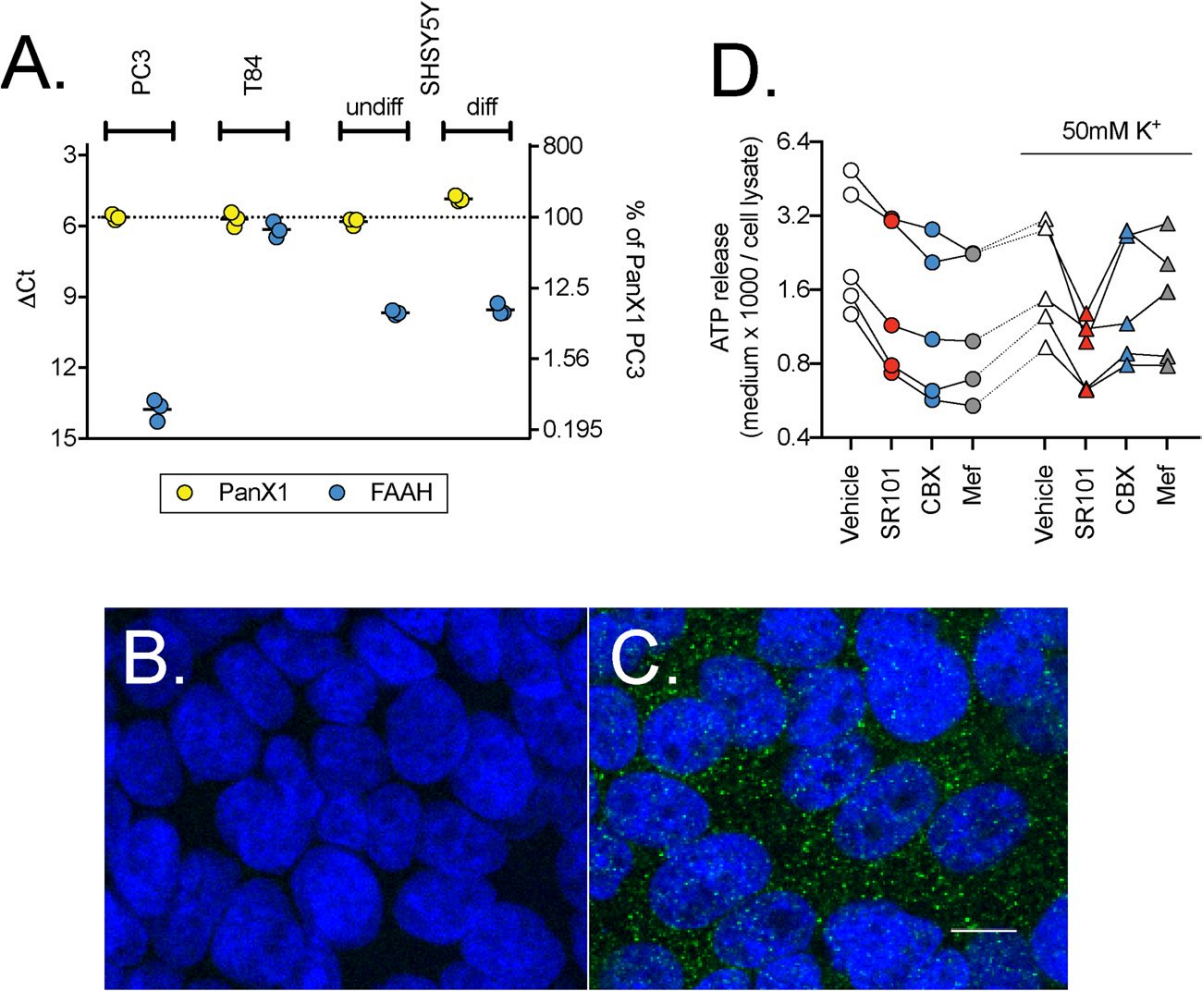
Expression of Panx1 in T84 cells. Panel A shows the expression at the mRNA level of Panx1 and FAAH from three separate experiments for T84, PC3, undifferentiated and differentiated SHSY5Y cells. Shown are scatterplots of the ∆Ct values, with the right axes showing the values as % of the Panx1 mRNA expression in PC3 cells as the positive control. Panels B and C show confocal images of Panx1 (green) and nuclear counterstaining with DAPI (blue) immunofluorescence (80-fold magnification; 40x objective with 2x zoom) in T84 cells in the absence (**B**) and presence (**C**) of the Panx1 primary antibody. Note that the brightness and contrast in the images shown in Panels B and C have been adjusted (automatic level adjustment in the Preview application for the Macintosh) to make the fluorescence signal easier to visualize. The white scale bar in C represents 10 µm. The original images are available in Supplementary Fig. S1. Panel D shows the release of ATP from T84 cells in the presence of SR101 (10 µM), CBX (30 µM) or Mef (30 µM) (N = 5). Data for each experiment are connected by the lines. A two-way randomised block ANOVA with Geisser-Greenhouse-adjusted degrees of freedom using the log_10_ ATP release (see methods for rationale) gave a significant interaction treatment x K^+^ (P = 0.0048) and so the data was reanalysed using a one-way randomised block ANOVA with Geisser-Greenhouse-adjusted degrees of freedom at each [K^+^]. At the normal [K^+^], the P value for treatment was <0.0001, and post-hoc Dunnett tests vs. vehicle control gave adjusted P values of 0.0051, 0.0010 and 0.0006 for SR101, CBX and Mef, respectively. At 50 mM [K^+^], the P value was 0.010, and the post-hoc Dunnett tests vs. vehicle control gave adjusted P values of 0.029, 0.15 and 0.31 for SR101, CBX and Mef, respectively. At both [K^+^], the P value for the matching was <0.0001, indicating that the individual control and treatment values are correlated.

The expression of Panx1 and FAAH mRNA were determined by qPCR in T84, PC3 and SH-SH5Y cells. The expression level of Panx1 was similarly high in all three cell lines (Fig. 1A). In contrast, expression of FAAH varied considerably, with a low ~2^8^ lower levels (2^(−∆∆Ct)^) for PC3 cells compared to T84 cells. The low expression of FAAH in PC3 cells is consistent with the literature^34^. Panx1 is functionally expressed in PC3 and SH-SY5Y cells^32,33^. In order to confirm that Panx1 is also expressed at the protein level in T84 cells antibody labeling experiments were undertaken. No immunofluorescence signal was observed with omission of the primary antibody (Fig. 1B). Punctate staining was observed in/on the cells and the distribution was rather different to that seen with the nuclear marker counterstain DAPI (Fig. 1C). Note that the brightness and contrast of these two images have been digitally adjusted (automatic level adjustment in the Preview application for the Macintosh of the two images together) to make the expression easier to visualize; the unadjusted images are provided in Supplementary Fig. S1. Since the cells were grown as a monolayer, they produced tight junctions that were visualised with ZO1 immunoreactivity (Supplementary Fig. S2). Panx1 immunoreactivity did not overlap with the ZO1 indicating that it did not localize to tight junctions (Supplementary Fig. S2).

In order to assess whether or not Panx1 mRNA expression is reflected in functionally active channels, two approaches were used. In the first set of experiments, T84 cells were exposed to 10 µM SR101, which is transported by Panx1 channels (q.v.^30^). The observed fluorescence signal was normalised to the fluorescence signal obtained by nuclear counterstaining with DAPI. We found that SR101 exposure led to its robust intracellular accumulation but that increase in fluorescence was not blocked by two compounds that inhibit the channel: mefloquine (Mef) and carbenoxolone (CBX) (Supplementary Fig. S3), suggesting that cellular accumulation of SR101 in T84 cells is not primarily achieved via Panx1 channels under the assay conditions used. A similar conclusion has been drawn for the accumulation of SR101 by hippocampal astrocytes^35^. AEA (1 and 10 µM) was also ineffective in reducing SR101 accumulation by T84 cells (Supplementary Fig. S3). However, under our standard uptake assay conditions, T84 cells released ATP in a manner that was significantly reduced by 10 µM SR101, 30 µM CBX and 30 µM Mef (Fig. 1D). Note that the extracellular medium used for the experiments contains fatty acid-free bovine serum albumin (BSA; 0.1%). CBX has a high affinity for BSA^36^, but the inhibition seen here suggests that the concentration of CBX was sufficient. We chose 30 µM as a maximum concentration to avoid the risk of non-specific effects on the plasma membrane, as has been seen for other lipophilic compounds and for AEA itself^37^. The mean % of control values (SD and 95% CI values in brackets) were: SR101, 63 (9.8; 51–75); CBX, 50 (7.1; 42–59); and Mef, 49 (6.4; 41–57). We also investigated the effects of the compounds at a high medium K^+^ concentration (50 mM, with the Na^+^ concentration reduced by the corresponding amount). Under these conditions, ATP release was significantly reduced by SR101 (to 54% of control; SD 18; 95% CI 32–75) but not by the other compounds (Fig. 1D).

### Effects of URB597 upon the uptake and hydrolysis of AEA in T84 cells

In order to assess the sensitivity of AEA and 2-AG uptake by T84 cells to pharmacological manipulation, we characterised the effects of URB597, a selective inhibitor of FAAH^38^. In preliminary experiments, the ability of URB597 to inhibit the hydrolysis of 100 nM [Et-^3^H]AEA by intact cells was assessed. The median (range, N = 3) FAAH activity as % of vehicle treated cells was 113 (85–117), 99 (96–104), 38 (27–43), 8.4 (5.3–12) and 6.9 (6.7–7) for 0.1, 1, 10, 100 and 1000 nM URB597 respectively, thereby confirming that the expressed FAAH is active. Consistent with the ability of FAAH to regulate AEA uptake in cells^22,34^, the uptake of 100 nM [Ara-^3^H]AEA in T84 cells treated with vehicle and 1 µM URB597 were 2.68 ± 0.44 and 1.47 ± 0.37 pmol per well, respectively (means ± SD, N = 7; P < 0.001, two-tailed ratio paired-t-test). The corresponding values were 2.98 ± 0.63 and 1.46 ± 0.36, respectively, for undifferentiated SH-SY5Y cells (N = 8, P < 0.0001); 2.57 ± 0.48 and 1.35 ± 0.33 for 3 day differentiated SH-SY5Y cells (N = 8, P < 0.0001). Thus, inhibition of FAAH reduces the uptake of 100 nM AEA by about half. URB597 had a small, but significant effect on the retention of [Ara-^3^H]AEA by wells alone, with values of 0.47 ± 0.11 and 0.43 ± 0.11 pmol per well for vehicle and URB597-treated wells being found (N = 12, P = 0.0041). We have previously shown that [Ara-^3^H]AEA uptake by PC3 cells is not reduced by URB597, as expected since the expression of FAAH in these cells is low^34^. Thus, in the cells investigated here, the ability of FAAH to regulate [Ara-^3^H]AEA is dependent upon its expression.

### Effects of Panx1 inhibitors upon the uptake of AEA, PEA and 2-AG in T84 cells

If Panx1 is involved in the cellular accumulation of AEA under standard assay conditions (i.e. the normal K^+^ concentrations used in the ATP release experiments), then uptake of this endocannabinoid should be reduced by compounds that inhibit Panx1 channels. This has been investigated using Mef and CBX and ^10^Panx (with scrambled ^Scr^Panx as negative control)^39^ as well as with SR101 (for AEA and T84 cells). We have also investigated the effects of the Mef and CBX upon the uptake of PEA and 2-AG.

**Figure 2:**
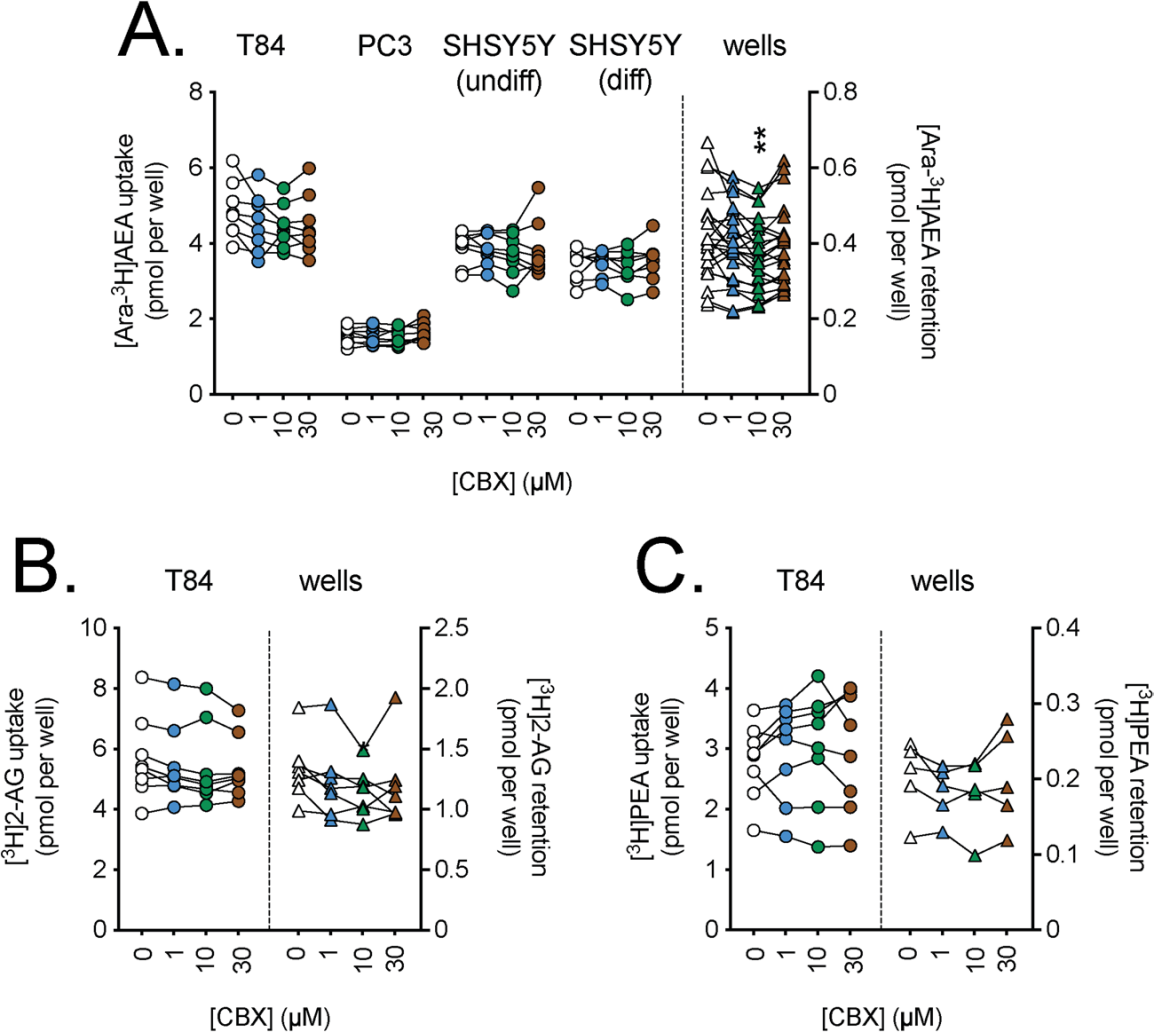
Effects of preincubation for 10 min with carbenoxolone (CBX) upon the uptake of (**A**), [Ara-^3^H]AEA; (**B**), [^3^H]2-AG and (**C**), [^3^H]PEA by T84 cells and the retention by wells alone. Data for each experiment and cell line are connected by the lines, with sample sizes as follows: Panel A, N = 8 (all cells) and 22 (wells); Panel B, N = 8 (cells) and 7 (wells); Panel C, N = 8 (cells) and 5 (wells). The data points for the cells relate to the left y-axes, and the data points for the wells relate to the right y-axes. One-way randomised block ANOVAe with Geisser-Greenhouse-adjusted degrees of freedom gave P values of Panel A: 0.079, 0.23, 0.53, 0.35 and 0.0045 for T84, PC3, undifferentiated SH-SY5Y, 3 day differentiated SH-SY5Y cells and wells, respectively; Panel B, 0.13 (T84 cells) and 0.073 (wells); Panel C, 0.50 (T84 cells) and 0.30 (wells). In all cases, the matching of the samples was significant (P < 0.0001). **P < 0.01 vs. vehicle controls, Dunnett’s multiple comparisons test following significant ANOVA.

**Figure 3:**
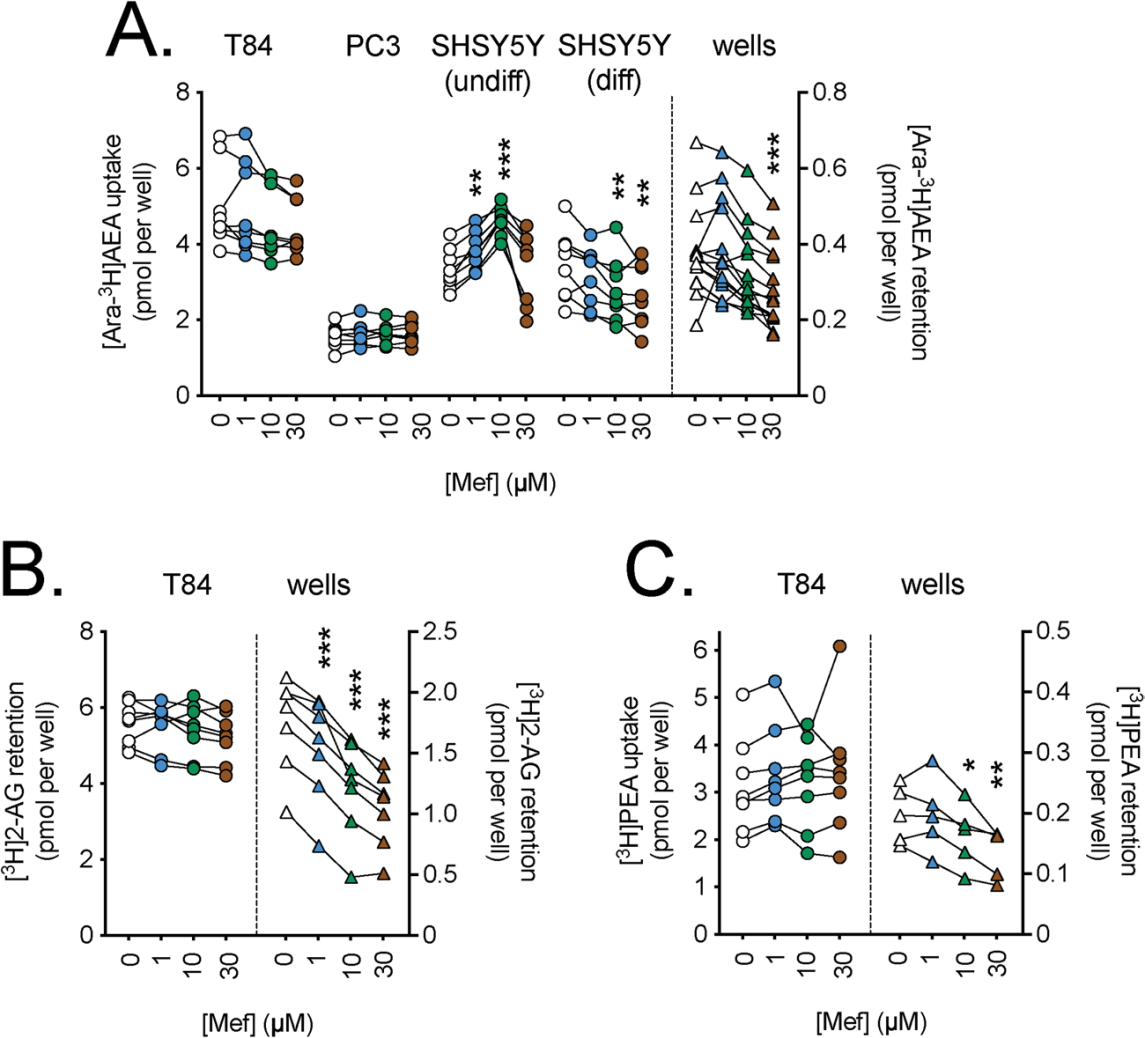
Effects of preincubation for 10 min with mefloquine (Mef) upon the uptake of (**A**), [Ara-^3^H]AEA; (**B**), [^3^H]2-AG and (**C**), [^3^H]PEA by T84 cells and the retention by wells alone. Data for each experiment and cell line are connected by the lines, with sample sizes as follows: Panel A, N = 8 (all cells) and 14 (wells); Panel B, N = 8 (cells) and 7 (wells); Panel C, N = 8 (cells) and 5 (wells). The data points for the cells relate to the left y-axes, and the data points for the wells relate to the right y-axes. One-way randomised block ANOVAe with Geisser-Greenhouse-adjusted degrees of freedom gave P values of Panel A: 0.080, 0.34, 0.0017, 0.0002 and <0.0001 for T84, PC3, undifferentiated SH-SY5Y, 3 day differentiated SH-SY5Y cells and wells, respectively; Panel B, 0.17 (T84 cells) and <0.0001 (wells); Panel C, 0.35 (T84 cells) and 0.0049 (wells). In all cases, the matching of the samples was significant (P ≤ 0.0002). *P < 0.05, **P < 0.01, ***P < 0.001 vs. vehicle controls, Dunnett’s multiple comparisons test following significant ANOVA.

**Figure 4:**
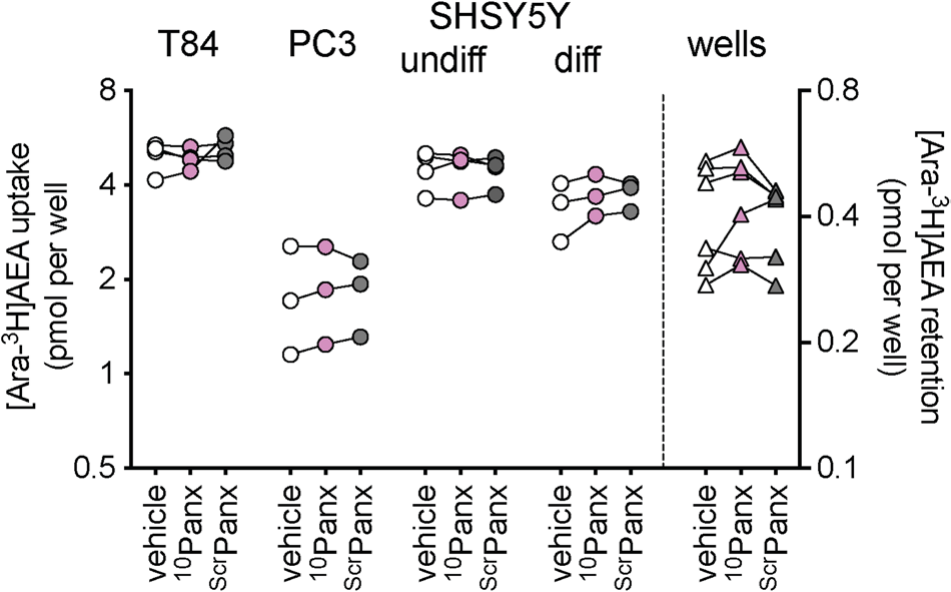
Effects of preincubation for 10 min with ^10^Panx and scrambled (Scr) Panx (both 100 µM) upon the uptake of 100 nM [Ara-^3^H]AEA by cells and the retention by wells alone. Data for each experiment and cell line are connected by the lines, N = 3–4. The data points for the cells relate to the left y-axes, and the data points for the wells relate to the right y-axes. One-way randomised block ANOVAe with Geisser-Greenhouse-adjusted degrees of freedom gave P values of 0.48, 0.69, 0.84, 0.18 and 0.26 for T84, PC3, undifferentiated SH-SY5Y, 3 day differentiated SH-SY5Y cells and wells, respectively.

**Figure 5:**
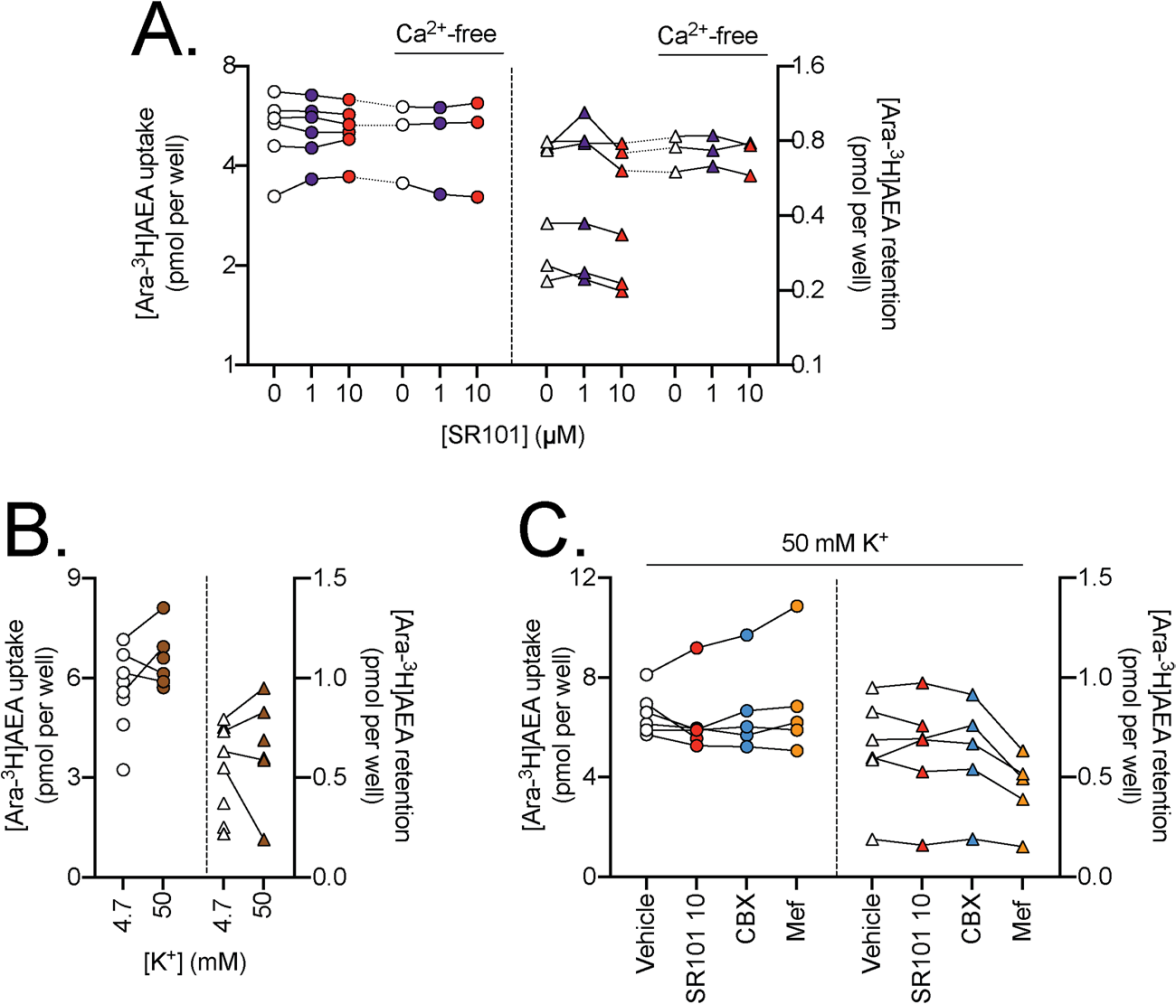
Panel A. Effect of preincubation for 10 min with SR101 upon the uptake of 100 nM [Ara-^3^H]AEA by T84 cells and the retention by wells alone, assays either in the presence or absence of Ca^2+^ in the medium. Data for each experiment and cell line are connected by the lines, N = 3–6. The data points for the cells relate to the left y-axes, and the data points for the wells relate to the right y-axes. One-way randomised block ANOVAe with Geisser-Greenhouse-adjusted degrees of freedom gave P values of: cells, 0.95 and 0.61; wells 0.051 and 0.57 for normal and Ca^2+^-free, respectively. The matching of the samples was significant (P ≤ 0.0034). Panel B. Comparison of 100 nM [Ara-^3^H]AEA in T84 cells and retention by wells for the assay [K^+^] shown. Matched data are shown (4 pairs out of 10 experiments). P values, calculated using a partially overlapping t-test, were: cells, 0.086; wells, 0.30. Panel C. Effect of preincubation for 10 min with SR101 (10 µM), 30 µM CBX and 30 µM Mef upon the uptake of 100 nM [Ara-^3^H]AEA by T84 cells and the retention by wells alone, assays conducted at 50 mM K^+^ (N = 6 for vehicle and SR101, N = 5 for CBX and Mef). Mixed-effects analyses matching for the experiment gave P values for treatment of 0.36 and 0.18, for cells and wells, respectively. In both cases, the matching was significant (P < 0.0001).

In the figures, the uptake for the cells refer to the scales on the left y-axes, while the retention by wells alone refer to the scales on the right y-axes. As pointed out in the methods, we have used a randomised block ANOVA (i.e. where both experiment day and treatment are included as factors) since this is recommended when there is a day-to-day variation in the measured dependent variable^40^. In the case of CBX, no significant effects upon uptake by T84 cells were seen for any of the three lipids (Fig. 2). Similarly, no significant effects of Mef upon the uptake of [Ara-^3^H]AEA, [^3^H]2-AG or [^3^H]PEA by T84 cells were seen (Fig. 3). Significant effects of Mef upon the uptake of [Ara-^3^H]AEA by the SH-SY5Y cells were seen. However, these are hard to interpret in terms of Panx1 function, since a) for the undifferentiated cells, the effect is an increase, rather than a decrease; and b) for the differentiated cells, the reduction in uptake in the cells is matched by a similar reduction (in percentage terms as opposed to the absolute reduction) in the retention of [Ara-^3^H]AEA by the wells themselves. ^10^Panx was also without effect upon AEA uptake, although the experimental sample size was modest in these experiments (N = 3–4; Fig. 4). Finally, in a set of ten experiments, SR101 was investigated under different conditions (normal assay medium, Ca^2+^-free medium and 50 mM K^+^ in the medium (with the [Na^+^] reduced by the corresponding amount; CBX and Mef were also included in those experiments). The different experiments were not completely matched for the different conditions, so the data is given in three separate figures (Fig. 5A–C). No statistically significant effects of SR101 upon AEA uptake by T84 cells in normal or Ca^2+^-free medium (Fig. 5A); of the increased [K^+^] per se upon uptake (Fig. 5B); or of the effects of the Panx1 inhibitors upon the uptake at 50 mM K^+^ (Fig. 5C) were seen.

### Effects of mefloquine and carbenoxolone upon AEA hydrolysis by T84 cells

**Figure 6:**
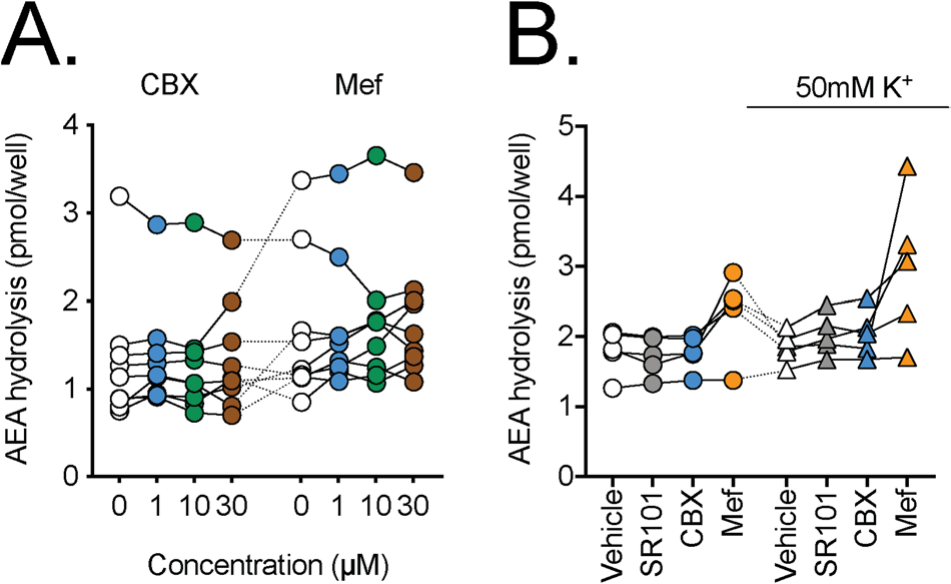
The effects of SR101, CBX or Mef upon the hydrolysis of 100 nM [Et-^3^H]AEA by T84 cells. The cells were preincubated for 10 min with test compounds or vehicle prior to addition of [Et-^3^H]-AEA and incubation for a further 15 min at 37 °C. Data for each experiment are connected by the lines. Panel A, concentration-response curves for CBX and Mef (N = 9). A randomised block ANOVAe with Geisser-Greenhouse-adjusted degrees of freedom gave P values of: 0.66 and 0.37 for CBX and Mef, respectively. In both cases, the matching was significant (P < 0.0001). In Panel B, concentrations used were 10 µM for SR101, 30 µM for CBX and 30 µM for Mef (N = 5). A two-way randomised block ANOVAe with Geisser-Greenhouse-adjusted degrees of freedom gave P values of: test compound, 0.013; K^+^ concentration, 0.47; test compound x K^+^ concentration 0.055.

If Panx1 is responsible for the transport of AEA in T84 cells, then the hydrolysis of exogenously administered [Et-^3^H]AEA, which is dependent upon the uptake of AEA into the cell, should be inhibited by CBX, Mef and SR101. In order to investigate this possibility, intact T84 cells were preincubated for 10 min with these compounds followed by addition of 100 nM (final concentration) of [Et-^3^H]AEA and incubation for a further 15 min. The assay buffer contained either normal or raised K^+^ concentrations. The data are shown in Fig. 6A,B for two separate sets of experiments. No significant effects of either CBX or SR101 were seen, whilst the effects of Mef were rather inconsistent.

### Effects of mefloquine and carbenoxolone upon the release of AEA from T84 cells

**Figure 7:**
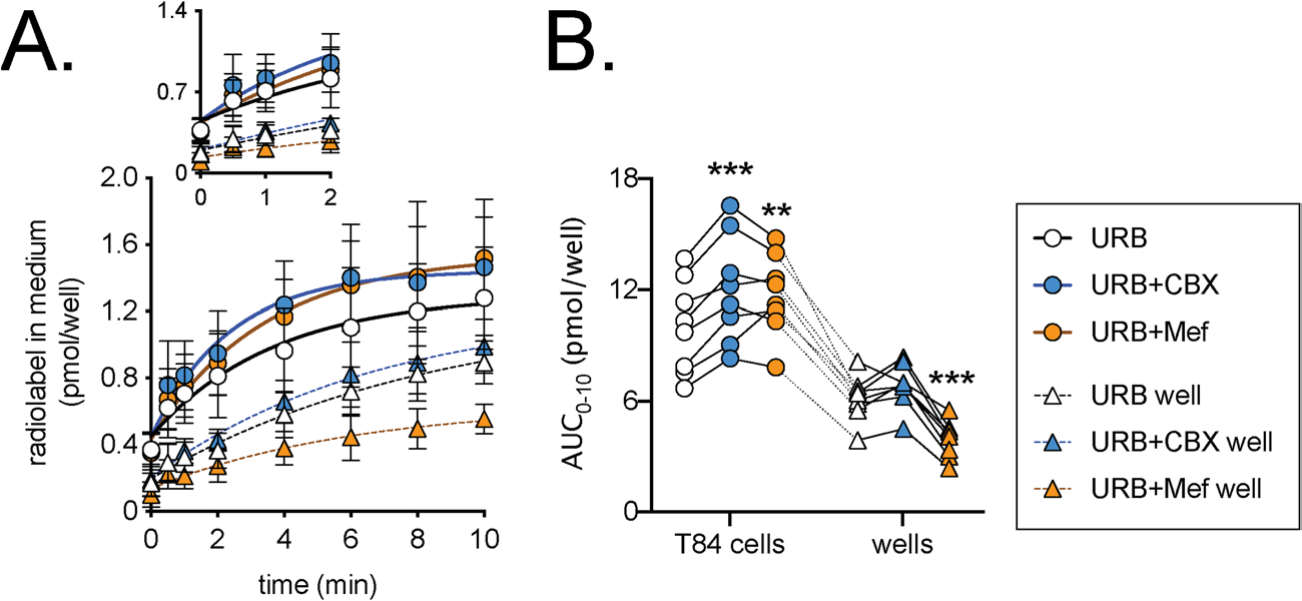
Inhibition of the release of tritium into the medium following incubation of T84 cells with 100 nM [Ara-^3^H]AEA in the presence of 1 µM URB597. In Panel A, cells or wells alone were preincubated for 10 min with 1 µM URB597 followed by incubation for 10 min with 100 nM [Ara-^3^H]AEA. The samples were then washed three times and then incubated in medium containing URB597 ± CBX or Mef and samples of the medium were taken over the next ten minutes. For Panel A, the data are means ± SD, N = 8. The radiolabel in medium is calculated taking into account the amount already released and the volume of medium remaining (see Methods). The inset in Panels A shows the data for the first 2 minutes. In Panel B, the individual areas under the curve for 10 min period studied (AUC_0–10_) are shown. Data for each experiment and “source” (cell or well) are connected by the lines (N = 8). A two-way randomised block ANOVAe with Geisser-Greenhouse-adjusted degrees of freedom gave P < 0.001 for test compound, source and for the interaction test compound x source. In view of the significant interaction, one-way randomised block ANOVAe with Geisser-Greenhouse-adjusted degrees of freedom were calculated for the cells and wells separately. Both showed significant P values (P ≤ 0.0006) and significant matching of the samples (P ≤ 0.0002). **P < 0.01, ***P < 0.001 vs URB alone, Dunnett’s multiple comparisons test.

Cells were preincubated with 1 µM URB597 and then labelled by incubation with 100 nM [^3^H]AEA after which they were extensively washed to minimise the extracellular label left in the medium. The release of tritium into the medium in the absence or presence of 30 µM CBX or 30 µM Mef were followed over 10 min (Fig. 7A). As reported previously^41^, the label retained by wells alone is released into the medium, but the initial rate from the cells was faster. The total release of label by the cells over the 10 min period was significantly increased by CBX and Mef (Fig. 7B). The effect of CBX was greater than Mef, but this may reflect the ability of Mef to reduce the release of label from the wells alone (Fig. 7B).

## Discussion

The present series of experiments were motivated by the findings of Hill, Thompson and colleagues that in the hippocampus Panx1 can act as a transporter for AEA^29,30^. Our approach has been purely pharmacological – to determine whether or not inhibitors of Panx1 channels affect the uptake of AEA, 2-AG and PEA. We were unable to detect any effects of the compounds in the T84 cells despite their expression of Panx1 and despite the ability of the compounds to reduce basal ATP release from the cells assayed using the same assay medium. Furthermore, we saw no effects of the Panx1 inhibitors on AEA uptake in either PC3 or SH-SY5Y cells, which have been shown to express functional Panx1 channels^33,34^. Thus, under the conditions used in our experiments, Panx1 does not contribute to the uptake of AEA (or 2-AG and PEA in the T84 cells) to an observable extent.

It is important to stress that our findings do not disprove the hypothesis that Panx1 can transport AEA, merely that in our cells under the assay conditions used, the contribution of such a mechanism to the uptake (and release) of AEA is negligible. In this respect, it is worth noting that C6 glioma cells do not express Panx1^42^, but can accumulate and hydrolyse extracellularly administered AEA^43,44^.

There are two interesting potential explanations for the discrepancy between the pronounced role of Panx1 in the hippocampus and the lack of a clear role in the cells investigated here. First, it is possible that the Panx1 transport route is a rapid phenomenon and that the conditions used in our uptake assays miss its contribution. However, a Panx1-mediated rapid transport of AEA would also be expected to be saturable, but the rapid cellular uptake of AEA (25 sec incubations) by N18TG2 neuroblastoma cells is not saturable^45^. In any case, the increase in uptake of the AEA analogue SKM 4-45-1 produced by transfection of HEK293 cells with Panx1 in^30^ was observable over a 20-minute incubation.

An alternative explanation is that while Panx1 is capable of transporting AEA, its capacity is limited and thus in the T84 cells, PC3 cells and SH-SY5Y cells, other mechanisms of accumulation dominate. We and others have argued that simple diffusion of the highly lipophilic AEA molecule across the plasma membrane followed by carrier-mediated intracellular transport to its intracellular targets and metabolic enzymes is sufficient to explain the properties of AEA uptake (temperature dependency and saturability)^45–48^. The hippocampal data of Hill, Thompson and colleagues^30^ would argue against this, simply because if passive diffusion dominates in our experiments, it would have been expected to dominate in the hippocampus as well, unless the distribution of Panx1 is asymmetric, i.e. is sufficiently large in a given cellular region that it can provide a localised transport of AEA. This may well be the case: in rodent hippocampal and cortical principal neurons, the distribution of Panx1 is asymmetric, co-localising with postsynaptic density protein 95^49^. Certainly, the subcellular distribution of endogenous Panx1 immunoreactivity varies considerably between cells^16^. Thus, in MDCK cells, Panx1 immunoreactivity is seen on the cell surface whereas in MC3T3-E1 cells the immunoreactivity is intracellular in nature^50^. In primary astrocyte and neuronal cultures, Panx1 immunoreactivity shows a punctate staining similar to our staining, but no obvious labelling was observed following cell-surface biotinylation^51^, suggesting that the majority of the Panx1 is intracellular in these primary cultures. We have not definitively assigned a cellular localisation to our Panx1 immunoreactivity using this type of experiment, since a negative result is not definitive evidence of absence, merely that expression upon the cell surface, if any, is below the limit of sensitivity of the assay. We regard our ATP release data as evidence of at least some expression of surface Panx1 channels. The function of intracellularly located Panx1 (other than its role in the lifecycle of the protein)^16^ is not yet clear, although it has been suggested that it may be involved in calcium release from endoplasmic reticuli^52^. If, for the sake of argument, it is suggested that the Panx1 in T84 cells is primarily intracellular, then our data would argue against a role of the protein in the intracellular trafficking of AEA akin to that described, for example, for fatty acid binding protein 5^23^. Indeed, in our assay system, a fatty acid binding protein 5 inhibitor significantly reduces the cellular uptake of AEA by several cell lines^53^.

In conclusion, we report that in T84 cells, concentrations of SR101, CBX and Mef that are capable of reducing ATP release do not reduce AEA uptake or its hydrolysis by intact cells, arguing against a major role of Panx1 in these cells in the uptake of AEA across the plasma membrane and/or its subsequent intracellular transport. As pointed out above, this does not reject the hypothesis that Panx1 can transport AEA in other cells, or that there is more to AEA transport than simple diffusion. In this respect, Chicca *et al*.^54^ have recently reported the ability of (2*E*,4*E*)-*N*-[2-(3,4-dimethoxyphenyl)ethyl]dodeca-2,4-dienamide potently to inhibit AEA uptake at a concentration three orders of magnitude lower than required for FAAH inhibition, to bind saturably to membranes from mouse brain and U937 macrophage cells, and to produce effects in models of pain and inflammation that were blocked by a CB_1_ receptor inverse agonist. Clearly, identification of the binding site for this compound will further the debate as to the nature of the elusive AEA plasma membrane transporter.

## Materials and Methods

### Materials

[Arachidonyl 5,6,8,9,11,12,14,15-^3^H]AEA ([Ara-^3^H]-AEA, for uptake and release experiments, specific activity 7.4 TBq mmol^−1^), [ethanolamine-1-^3^H]AEA ([Et-^3^H]AEA, for hydrolysis experiments, specific activity 2.2 TBq mmol^−1^), [arachidonoyl 5,6,8,9,11,12,14,15-^3^H]2-AG (for uptake experiments, specific activity 7.4 TBq mmol^−1^) and [palmitoyl 9,10-^3^H]PEA (for uptake experiments, specific activity 2.2 TBq mmol^−1^) were obtained from American Radiolabeled Chemicals Inc., St Louis (MO, USA). Mefloquine, carbenoxolone and sulforhodamine 101 (SR101) were obtained from Sigma-Aldrich Inc., St Louis (MO, USA). Non-radioactive AEA, PEA, 2-AG and URB597 (3′-(aminocarbonyl)[1,1′-biphenyl]-3-3-yl)-cyclohexylcarbamate) were obtained from the Cayman Chemical Co., Ann Arbor, (MI, USA.). ^10^Panx and Scrambled ^10^Panx were obtained from Tocris Bioscience. Rabbit anti human Panx1 antibody, anti-ZO-1 (ZO1-1A12), goat anti-rabbit Alexa fluor 488, Alexa Fluor 594 and DAPI were purchased from Life Technologies, Thermo Fisher Scientific, Waltham (MA USA).

### Cell cultures

All cells were grown in 75 cm^2^ flasks at 37 °C under humidified atmospheric pressure and supplemented with 5% (v.v^−1^) CO_2_. Cells were split into new flasks once or twice per week. For the uptake and hydrolysis experiments, the cells (2.5 × 10^5^ cell per well for T84 and SH-SY5Y, 10^5^ cells per well for the larger PC3 cells) were plated into 24 well culture plates and cultured overnight in humidified air at 37 °C with 5% CO_2_. The T84 cell line (American Type Culture Collection, Rockville, MD, USA) was a gift from Prof. Marie-Louise Hammarström (Department of Clinical Microbiology, Umeå University). These were cultured in DMEM/F12 medium (Thermo Fisher Scientific, Waltham, MA, USA), 2 mM L-glutamine, 8% (v.v^−1^) foetal bovine serum and 1% penicillin/streptomycin (PEST). PC3 human prostate cancer cells were obtained from DSMZ (Braunschweig, Germany) and cultured in Ham’s F-10 medium (Thermo Fisher Scientific, Waltham, MA, USA), 2 mM L-glutamine, 10% foetal bovine serum and 1% PEST. SH-SY5Y human neuroblastoma cells were purchased from European Collection of Authenticated Cell Cultures (Porton Down, UK). They were cultured in minimal essential medium with Earl´s salts (EMEM) supplemented with 10% FBS, 1% NEAA (non-essential amino acids) supplement, 2 mM L-glutamine and 1% PEST. Differentiation of SH-SY5Y cells was undertaken by replacing the medium with a differentiation medium (Dulbecco’s modified medium with Ham´s F12 medium (1:1), 1% N2 supplement, 1 µM retinoic acid and 1% PEST). The cells were differentiated for 3 days, and half of the medium in each well was changed every 48 h. Retinoic acid-differentiated human neuroblastoma SH-SY5Y cells have a prominently dopaminergic-like neuronal phenotype^55^ and thus are convenient model for neuronal AEA uptake. In the methods described below, concentrations in % are w.v^−1^ or v.v^−1^, as appropriate.

### mRNA quantification

mRNA extraction was performed using a Dynabeads mRNA DIRECT kit (Thermo Fisher Scientific, Waltham, MA, USA). Briefly, medium was removed from the 24-well plate, cells were gently washed with phosphate-buffered saline (PBS) and then suspended in 300 μL of lysis buffer; after multiple passages through a pipette tip to obtain complete lysis. Lysate was frozen and stored at −80 °C. mRNA was then extracted using the Dynabeads mRNA direct kit according to the manufacturer’s instructions. After quantification (Nanodrop Lite®, Thermo Fisher Scientific), the extracted mRNA was diluted in TE buffer to a concentration of 5 ng μL^−1^ (10 µL total) and mixed with the 2XRT master mix prepared as described in the high capacity cDNA reverse transcription kit (Thermo Fisher Scientific). cDNA was diluted 1/10 and loaded into the assay plates. qPCR was performed with a Eco Real-Time PCR system (Illumina Inc., San Diego, CA). PCR reactions were run on each sample in duplicate using KAPA SYBR FAST qPCR kit Master Mix with the following conditions for amplification: an initial holding stage of 3 min at 95 °C, then 45 cycles consisting of denaturation at 95 °C for 3 s and annealing/extension at 60 °C for 30 s. Products were analysed by performing a melting curve at the end of the PCR reaction. Data were normalized to the 60S ribosomal protein L19 (RPL19). Shown in graphs is the ΔCt (i.e. threshold cycle (Ct) of the gene of interest – Ct of the reference gene RPL19). The primers used are given in Table 1.

**Table 1:** Primers used in the present study.

Gene	Product	Forward primer (5′ to 3′)	Reverse primer (5′ to 3′)
*FAAH*	FAAH	CACACGCTGGTTCCCTTCTT	GGGTCCACGAAATCACCTTTGA
*PANX1*	PANX1	GCGTGACCTTGACATGAGAG	TACCTCCCACAAACTTTGCC
*RPL19*	RPL19	CACATCCACAAGCTGAAGGCA	CTTGCGTGCTTCCTTGGTCT

### Immunofluorescent labeling of Panx1

T84 cells were plated on inserts (2.5 × 10^5^ cells per insert) and allowed to attach. One day later, inserts were washed with PBS and fixed for 15 minutes at room temperature with 4% paraformaldehyde in PBS and then washed twice with PBS and twice with PBS containing 0.1% BSA before being incubated for 30 minutes at room temperature in blocking buffer (PBS with 5% BSA and 0.3% Triton X100). After 3 washes with PBS, the inserts were incubated for 1 hour at room temperature with or without (no primary control) the Panx1 antibody (1/1000) diluted in PBS with 1% BSA and 0.3% Triton-X100. Following three washes with PBS, the inserts were incubated with the secondary antibody (Alexa Fluor 488, 1/250), diluted in the same buffer as the primary, for 1 hour at room temperature. Inserts were washed three times in PBS and DNA was labelled with 50 ng mL^−1^ DAPI for 5 minutes at room temperature. After two washes in PBS, the membranes were cut and mounted using Vectashield® HardSet. Images (512 × 512 pixels) were acquired using a Nikon A1 confocal microscope equipped with a 40x water immersion lens (CFI Apo Lambda S, NA 1.25) at Nyqvist resolution as a single XY plane (20.5 μm pinhole, 2 scanner zoom) or a Z-stack (Step = 0.2 μm), which was used to generate maximum intensity projections using NIS Elements software (Nikon).

### ATP release from T84 cells

T84 cells were plated in 96 well plates (50 000 cells/well) and incubated overnight in DMEM/F12 medium supplemented with 2 mM L-glutamine, 8% foetal bovine serum and 1% penicillin/streptomycin (PEST). The next day, medium was discarded and the cells were washed twice with Krebs-Ringer Hepes (KRH) buffer (120 mM NaCl, 4.7 mM KCl, 2.2 mM CaCl_2_, 10 mM HEPES, 0.12 mM KH_2_PO_4_, 0.12 mM MgSO_4_ in milliQ deionised water, pH 7.4). The buffer was removed and the cells were incubated with 100 µL of KRH or KRH with 50 mM K^+^, both with 0.1% fatty acid-free BSA, containing SR101 (10 µM), carbenoxolone (30 µM), mefloquine (30 µM) or vehicle control (DMSO, maximum assay concentration 0.05%) for 15 minutes. ATP luminescence was measured using the CellTitre Glo (ref G7570) kit from Promega (Madison, WI 53711 USA). Briefly, aliquots (50 µL) of medium were then transferred to a new plate and 50 µL of the reagent were added before the plate was read on a BioTek Synergy 2 luminometer (BioTek, Winooski, VT, USA). Reagent (50 µL) was also added to the cells and luminescence was read following lysis. Luminescence in the medium was normalized to luminescence in the cells.

### AEA, PEA and 2-AG uptake

This assay was performed following the method used by Rakhshan *et al*.^56^ as modified by Sandberg *et al*.^57^. Briefly, cells were plated into 24-well culture plates and cultured overnight at 37 °C in humidified atmosphere with 5% CO_2_. The next day, cells were washed with 400 µL of KRH with 1% BSA and thereafter with 400 µL of KRH buffer alone, both solutions at 37 °C. The buffer was removed and then 350 µL of KRH with 0.1% fatty acid-free BSA and the compound of interest or vehicle control (DMSO, maximum assay concentration 0.1%) were added to the wells. After 10 minutes of incubation at 37 °C, 50 µL of [Ara-^3^H]AEA (with non-radioactive AEA added to give a 100 nM final concentration in KRH with 0.1% fatty acid-free BSA) was added to the wells; plates were incubated for further 15 minutes at 37 °C to allow AEA uptake into the cells. Subsequently plates were put on ice and washed three times with 500 µl of KRH with 1% BSA. Washing solution was removed, 500 µL of 0.2 M NaOH was added to lyse cells; plates were incubated for 15 minutes at 75 °C. Aliquots (300 µL) of the lysate solutions were taken into scintillation vials, 4 mL of scintillation liquid were added and the samples were analysed for tritium content by liquid scintillation spectroscopy with quench correction. Wells without cells were also assayed. The same protocol was used for 2-AG and PEA uptake but using 2-AG labelled on the arachidonate moiety and PEA labelled on the palmitoyl moiety, respectively.

### AEA hydrolysis

Cells were plated on 24-well plates, incubated overnight at 37 °C, washed before with KRH buffer with 1% BSA and then with KRH alone; then they were treated with the compound of interest diluted in 350 µL KRH with 0.1% fatty acid-free BSA and incubated for 10 minutes. Fifty µl of [Et-^3^H]AEA (with non-radioactive AEA added to give a final concentration of 100 nM), diluted in KRH with 0.1% fatty acid-free BSA were added to each well. After 15 minutes of incubation at 37 °C plates were put on ice and 600 µL/well of activated charcoal buffer (120 μL activated charcoal + 480 μL 0.5 M HCl) were added to separate product from substrate^53,58^. Aliquots of 600 µL were then transferred into glass tubes and centrifuged for 15 minutes at 2500 rpm. Aliquots of 200 µL of supernatant (containing the [^3^H]ethanolamine product) were pipetted into scintillation vials, 4 ml of scintillation liquid were added and the samples were analysed for tritium content by liquid scintillation spectroscopy with quench correction. Blanks (wells not containing cells) were run concomitantly.

### AEA release

The protocol for AEA release was adapted from the protocol for AEA uptake. Cells were plated into 24-well culture plates and cultured overnight at 37 °C in humidified atmosphere with 5% CO_2_. The next day, cells were washed with 400 µL of KRH buffer with 1% BSA and then with 400 µL of KRH buffer alone. The buffer was removed and then 350 µL of KRH with 0.1% fatty acid-free BSA and URB597 (1 µM) were added to the wells. After 10 minutes of incubation at 37 °C, 50 µL of [Ara-^3^H]AEA (with non-radioactive AEA added to give a 100 nM final concentration) in KRH with 0.1% fatty acid-free BSA was added to the wells and an aliquot of 50 µL was taken. The plates were incubated for a further 15 minutes at 37 °C to allow AEA uptake into the cells. After the 15 minutes, the buffer was removed and the cells were washed three times in buffer containing vehicle (DMSO, maximum assay concentration 0.1%), 30 µM CBX or 30 µM Mef before being incubated again with 400 µL of buffer containing vehicle, CBX or Mef. Subsequently 20 µL samples were taken at t0 and the indicated time points and transferred to scintillation vials. 4 mL of scintillation liquid were added and the samples were analysed for tritium content by liquid scintillation spectroscopy with quench correction. Wells without cells were also assayed for comparative purposes. The release of radiolabel was calculated by taking into account both the radioactivity in the current sample, the volume reduction due to the sampling, and the amount of radioactivity already released. Thus, for example, a value at time 4 min is calculated as d.p.m. in sample × 320/400 + (d.p.m. at t0 × 20/400) + (d.p.m. at t0.5 × 20/400) + (d.p.m. at t1 × 20/400) + (d.p.m. at t2 ×20/400). Areas under the curve for the 10 min release period (AUC_0–10_) were calculated using the function auc (type = spline) in the package MESS v 0.5.5 for the Statistical Programme R v.3.5.1 for the Macintosh.

### Statistics

Throughout, the Ns given refer to the number of separate experiments. For the statistical analyses, two issues have been considered. Firstly, it has to be decided whether observed changes between control and treatment groups are likely to be proportional (i.e. a drop of, say, 25%) or incremental (i.e., a drop of, say, 0.5 units regardless as to the control levels). For an incremental effect of the test compound (which we have for simplicity assumed to be the case here for a Panx1 contribution to AEA uptake and its subsequent hydrolysis), then analysis of untransformed data is appropriate. For a proportional effect (which could be argued if the observed biological effect is brought about by a single target, and the parameter under study is the effect of an inhibitor of this target at a given concentration), then analysis of log data is more appropriate, since the difference between control and treatment reflects the ratio of values (for a discussion with respect to two paired sets of values, see^59^). Secondly, there was a large inter-experimental variability in our experiments which could mask treatment effects. In other words, the control and treatment values are correlated rather than independent. In his simulated experiment with two treatments upon cultured cells where the data was correlated due to a day-to day variation in the values, Lew^40^ demonstrated that a randomised block ANOVA (i.e. where both experiment day and treatment are included as factors) was more powerful than a standard one-way ANOVA. He recommended that “where there is, or might reasonably be, such a correlation (e.g. relatedness among the data within a single experimental run, or within a multi-well culture plate, or within an animal, et cetera), use the more powerful randomized block ANOVA rather than one-way ANOVA^40^”, and we have followed this recommendation. Additionally, we have presented the data as scatterplots with each matched sample set being identified. The randomised block ANOVAe (termed one-way repeated measures ANOVA in the programme used) not assuming sphericity (Geisser-Greenhouse-adjusted degrees of freedom) were conducted using the GraphPad Prism 8.0.2 programme for the Macintosh (GraphPad Software Inc., San Diego, CA). In one set of experiments, not all data was matched and so these have been analysed either by a partially overlapping t-test^60^ (function Partover.test in the package Partiallyoverlapping v2.0 for the R statistical programme^61^ v. 3.5.1; parameter var.equal set to FALSE), or by a mixed effects model (REML) not assuming sphericity in the GraphPad Prism programme, as appropriate.

## Supplementary information

Supplementary Information

## Data Availability

The datasets generated during the current study are available from the corresponding author on reasonable request.
