# Supplementary material for: Role of pannexin-1 in the cellular uptake, release and hydrolysis of anandamide by T84 colon cancer cells
Source: Sci Rep. 2019 May 20;9:7622. doi: 10.1038/s41598-019-44057-x (PMC6527687; doi:10.1038/s41598-019-44057-x)
Supplement: Supplementary file 1 — Supplementary Information [file 41598_2019_44057_MOESM1_ESM.docx]

**Supplementary Information**

**Role of pannexin-1 in the cellular uptake, release and hydrolysis of anandamide by T84 colon cancer cells**

**Mireille Alhouayek, René Sorti, Jonathan D. Gilthorpe & Christopher J. Fowler**

**
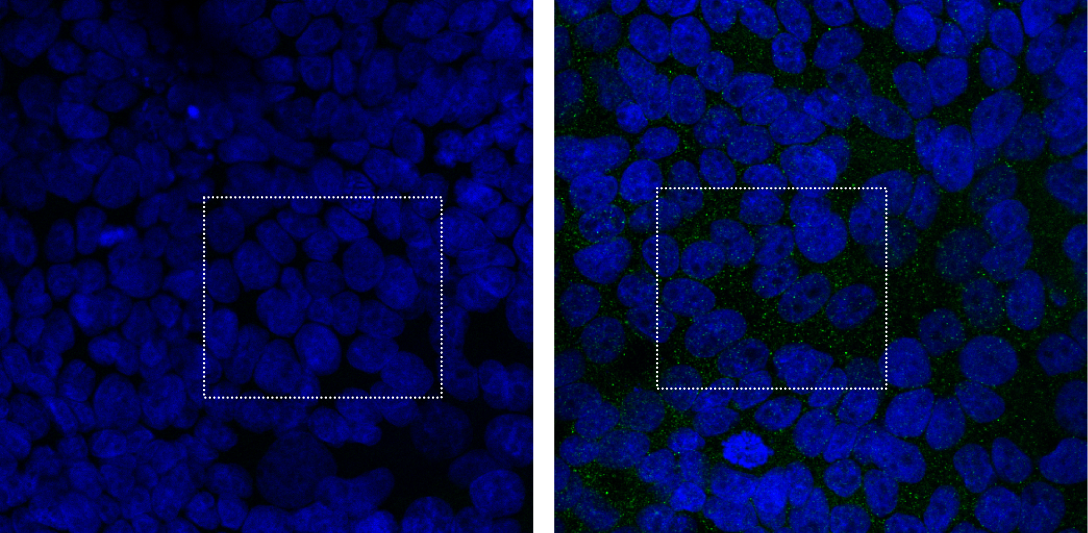
**

**Supplementary Fig. S1.** Original confocal images of immunofluorescence labeling shown in Fig. 1B (left) and C (right) of the paper. The white rectangles show the parts of the image that were cropped and used in the main figure. Images (512x512 pixels, 0.31 μm/pixel) were acquired using a Nikon A1 confocal microscope equipped with a 40x water immersion lens (CFI Apo Lambda S, NA 1.25) at Nyqvist resolution (525/50 nm for PANX1 labelling in green, 20.5 μm pinhole, 2.000 scanner zoom) in X and Y dimensions.


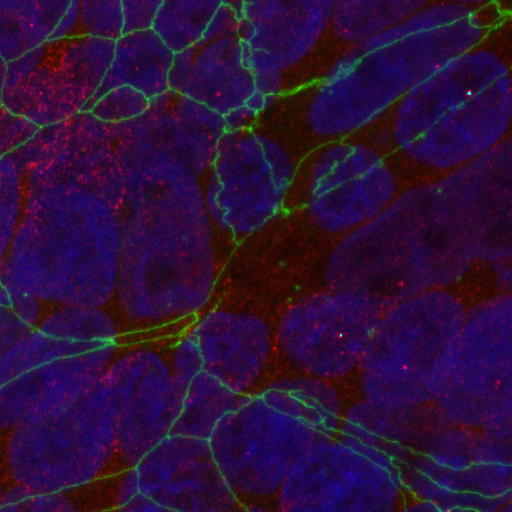


**Supplementary Fig. S2.** Expression of Panx1 (red) and the tight junction marker ZO-1 (green) in T84 cells, counterstained with DAPI (blue). The same protocol was used as for the immunofluorescent labeling method described in the main paper up to and including the incubation with the Panx1 antibody. Thereafter, following three washes with PBS, inserts were incubated with the secondary antibody (Alexa Fluor 594, 1/1000), diluted in the same buffer as the primary antibody, for 1 hour at room temperature. The inserts were then washed three times in PBS and incubated for 1 hour at room temperature with the anti-ZO1 antibody coupled to Alexa Fluor 488 (1/500). After three washes in PBS, DNA was labelled with 50 ng mL-1 DAPI for 5 minutes at room temperature. Inserts were washed twice with PBS and the membranes were cut and mounted using Vectashield® HardSet. Images (512x512 pixels) were acquired using a Nikon A1 confocal microscope equipped with a 40x water immersion lens (CFI Apo Lambda S, NA 1.25) at Nyqvist resolution (525/50 nm for ZO1 labelling in green, 595/59 nm for PANX1 labelling in red, 21.7 μm pinhole, 4.878 scanner zoom) in X, Y and Z dimensions. A Z-stack (22 steps with Step = 0.2 μm) was used to generate a maximum intensity projections using NIS Elements software (Nikon).

**Supplementary method:**

**Sulforhodamine 101 uptake:** T84 cells (15000cells/well) were plated in a 96 well plate and incubated overnight in DMEM/F12 medium supplemented with 2mM L-glutamine, 8% foetal bovine serum and 1% penicillin/streptomycin (PEST). The next day, cells were washed with 200 µL of KRH buffer with 1% BSA and thereafter with 200 µL of KRH buffer alone, both at 37 °C. The buffer was removed and then 225 µL of KRH, or KRH with 50mM K^+^, with 0.1% fatty acid-free BSA and the inhibitor of interest (Carbenoxolone or Mefloquine, both at 30µM) or vehicle control (DMSO, maximum assay concentration 0.1%) were added to the wells. After 10 minutes of incubation at 37°C, 25µL of sulforhodamine 101 (10µM final concentration in KRH or KRH with 50mM K^+^, with 0.1% fatty acid-free BSA buffer) were added to all the wells (except the control wells). Plates were incubated for further 10 minutes at 37 °C to allow sulforhodamine 101 uptake into the cells. Subsequently plates were put on ice and washed three times with 250 µl of KRH with 1% BSA. Washing solution was removed and 300 µL of KRH with 0.1% fatty acid-free BSA were added to the wells. The plate was then imaged using TROPHOS Plate RUNNER HD cell fluorescence imaging instrument (currently marketed as Dioscure HD Imager, Dioscure, Marseille, France). Total cell fluorescence from sulforhodamine 101 was determined using the Tina analysis package provided by the manufacturer. After imaging, the buffer was removed and the plate washed with PBS, fixed with 4% formaldehyde and then stained with 0.1µM DAPI. The plate was then imaged again using TROPHOS Plate RUNNER HD and total cell fluorescence from DAPI was determined using the Tina analysis package. Sulforhodamine 101 fluorescence was then normalized to DAPI fluorescence for each well


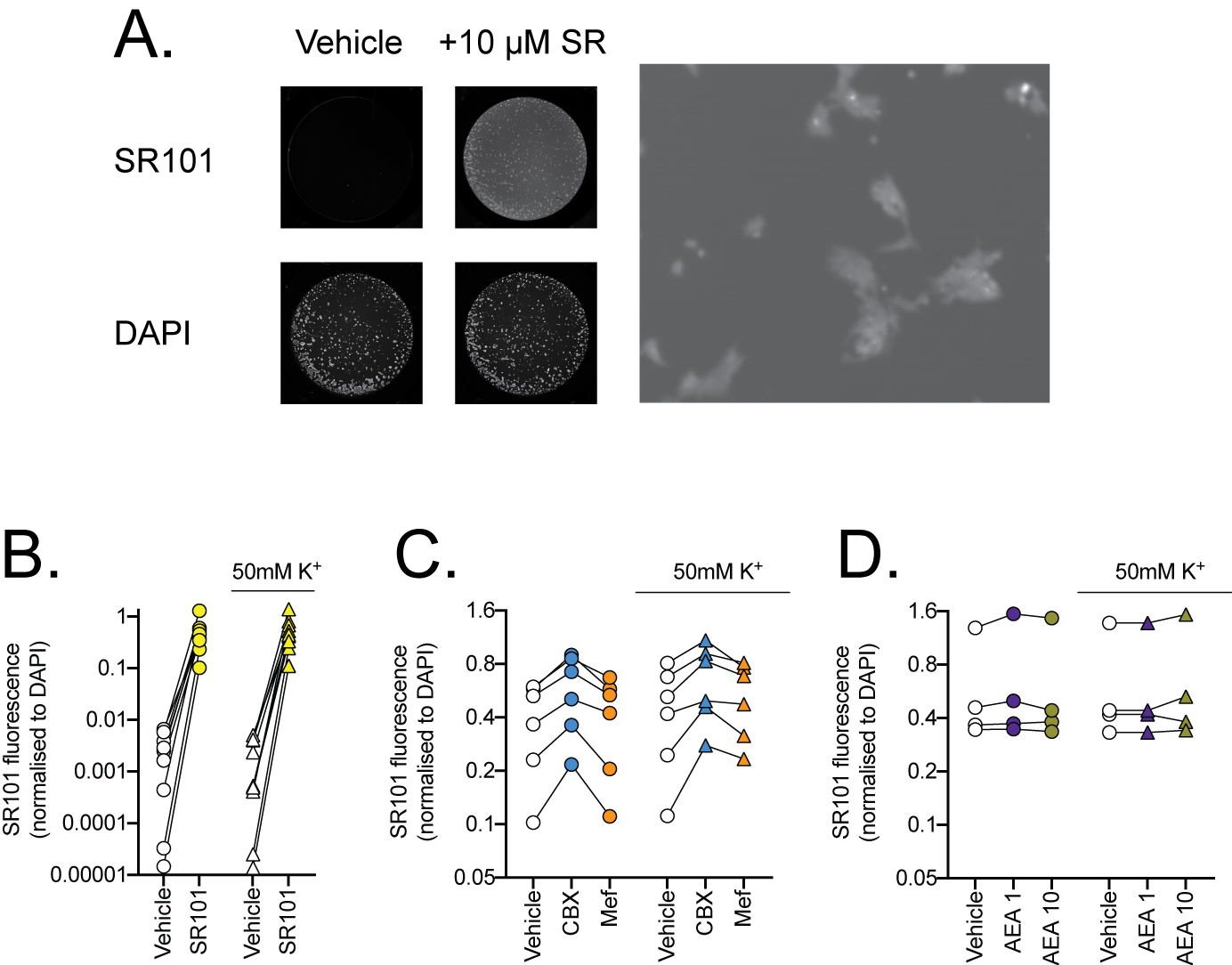


**Supplementary Figure S3.** Cellular uptake of SR101 by T84 cells. Panel A shows low resolution photographs of the wells, with fluorescence of either SR101 (top) or DAPI (bottom). The wells were incubated with either vehicle or 10 µM SR101. The right panel shows a higher resolution of SR101 fluorescence from another experiment, where the intracellular fluorescence can clearly be seen. Panels B-D show SR101 fluorescence normalised to the DAPI fluorescence. The lines connect data from the same experiment at each K^+^ concentration. Note that the y-axes are log scale. For Panel C, a two-way repeated measures ANOVA gave P values of 0.00086, 0.0062 and 0.087 for effect of treatment, K^+^ and treatment x K^+^, respectively. For Panel D, P values of 0.057, 0.019 and 0.82 for the effects of AEA, K^+^ and AEA x K^+^, respectively, were found.
